# Core metabolism plasticity in phytoplankton: Response of *Dunaliella tertiolecta* to oil exposure

**DOI:** 10.1111/jpy.13286

**Published:** 2022-09-29

**Authors:** Manoj Kamalanathan, Savannah Mapes, Alexandra Prouse, Patricia Faulkner, Nathan Hagen Klobusnik, Jessica Hillhouse, David Hala, Antonietta Quigg

**Affiliations:** ^1^ Department of Marine Biology Texas A&M University at Galveston Galveston Texas 77573 USA; ^2^ Department of Oceanography Texas A&M University College Station Texas 77845 USA; ^3^ Present address: Bigelow Laboratory for Ocean Sciences East Boothbay Maine 04544 USA; ^4^ Present address: Virginia Institute of Marine Science Gloucester Point Virginia 23062 USA

**Keywords:** inhibitors, Kreb's cycle, oil, pathway, photorespiration, phytoplankton

## Abstract

Human alterations to the marine environment such as an oil spill can induce oxidative stress in phytoplankton. Exposure to oil has been shown to be lethal to most phytoplankton species, but some are able to survive and grow at unaffected or reduced growth rates, which appears to be independent of the class and phylum of the phytoplankton and their ability to consume components of oil heterotrophically. The goal of this article is to test the role of core metabolism plasticity in the oil‐resisting ability of phytoplankton. Experiments were performed on the oil‐ resistant chlorophyte, *Dunaliella tertiolecta*, in control and water accommodated fractions of oil, with and without metabolic inhibitors targeting the core metabolic pathways. We observed that inhibiting pathways such as photosynthetic electron transport (PET) and pentose‐phosphate pathway were lethal; however, inhibition of pathways such as mitochondrial electron transport and cyclic electron transport caused growth to be arrested. Pathways such as photorespiration and Kreb's cycle appear to play a critical role in the oil‐tolerating ability of *D. tertiolecta*. Analysis of photo‐physiology revealed reduced PET under inhibition of photorespiration but not Kreb's cycle. Further studies showed enhanced flux through Kreb's cycle suggesting increased energy production and photorespiration counteract oxidative stress. Lastly, reduced extracellular carbohydrate secretion under oil exposure indicated carbon and energy conservation, which together with enhanced flux through Kreb's cycle played a major role in the survival of *D. tertiolecta* under oil exposure by meeting the additional energy demands. Overall, we present data that suggest the role of phenotypic plasticity of multiple core metabolic pathways in accounting for the oxidative stress tolerating ability of certain phytoplankton species.

**Abbreviations**
AOA: aminooxyacetate
CET: cyclic electron transport of PSI
ConA: conclavin A
DBMIB: 2,5‐dibromo‐3‐methyl‐6‐isopropyl‐p‐benzoquinone
DCFH‐DA: 2′,7′‐dichlorofluorescien diacetate
DCMU: 3‐(3,4‐dichlorophenyl)‐1,1‐dimethylurea
FADH: reduced flavin adenine dinucleotide
*F*_v_/*F*_m_
: maximum PSII quantum yield
MET: mitochondrial electron transport
NADH: reduced nicotinamide adenine dinucleotide
NADPH: reduced nicotinamide adenine dinucleotide phosphate
PET: photosynthetic electron transport
PPP: pentose‐phosphate pathway
*r*ETR_max_
: relative electron transport rates
ROS: reactive oxygen species
TCA: Kreb's/tricarboxylic acid
WAF: water accommodated fraction of oil
*α*
: light harvesting efficiency
σPSII: functional absorption cross‐section of PSII
ρ: connectivity factor
τ: time constant of the relaxation kinetics of the fluorescence yield following the single turnover flash

Marine phytoplankton are responsible for nearly 50 pg C · y^−1^ in aquatic systems (Field et al. 1998) and play a significant role in influencing elemental cycles such as nitrogen, phosphorus, and silica (Benitez‐Nelson 2000, Zehr and Kudela 2011, Tréguer and De La Rocha 2013). Therefore, it is important to understand the effects of human alteration on the marine environment, especially those inducing oxidative stress on phytoplankton. The 2010 Deepwater Horizon oil spill exposed the Gulf of Mexico to nearly 4.9 million barrels of oil (Turner et al. 2014). As a means of remediation, nearly 2.9 million liters of a chemical dispersant Corexit was applied to progress dispersion of the oil (Kujawinski et al. 2011, Quigg et al. 2021a). Overall, this event exposed marine organisms to unfamiliar and potentially toxic conditions (Solo‐Gabriele et al. 2021). Exposure to oil has previously been shown to induce oxidative stress in phytoplankton (Ozhan et al. 2015, Kamalanathan et al. 2021a,b), with screening experiments conducted by Bretherton et al. (2018, 2020) demonstrating the range of responses exhibited by phytoplankton and even within the same species (e.g., *Skeletonema* sp.). Given the toxic nature of the hydrocarbon components of oil, it is not surprising that much of the phytoplankton community are sensitive to its exposure (DeLaune et al. 1990, Quigg et al. 2021b). Therefore, it is unexpected to see some species (e.g., the chlorophyte, *Dunaliella tertiolecta*) exhibit resistance to the exposure of oil and/or dispersant Corexit, raising the question about the mechanisms that allow them to tolerate or resist the exposure to oil.

The genomic diversity within phytoplankton species can vary immensely, for example, the nuclear DNA content of chlorophytes has been shown to range from ~100 to ~31,600 Mb (Gregory 2005). This in‐turn can introduce much variation in the occurrence and the levels of expression of certain biochemical pathways. Therefore, most of the variability in response to an oxidative‐stress‐inducing environment, such as oil exposure (*Thalassiosira pseudonana* being sensitive and *Phaeodactylum tricornutum* resistant; Bretherton et al. 2018, 2020, Kamalanathan et al. 2019, 2021a,b), may be simply explained by the differences in genome composition and hence their metabolic capability. However, the effect of oxidative‐stress‐inducing toxic conditions on the core metabolic pathways, such as photosynthetic electron transport (PET), cyclic electron transport (CET) of PSI, Kreb's cycle, mitochondrial electron transport (MET), pentose‐phosphate pathway (PPP), and photorespiration, that are ubiquitously present in all phytoplankton remains unclear. Core metabolic pathways perform the fundamental functions of generating and consuming energy (ATP and NADPH/NADH/FADH) and synthesizing building blocks for cellular multiplication. Flexibility in core metabolic pathways vary between species under different physiological conditions (Kramer and Evans 2011). Such phenotypic plasticity can allow a given species of phytoplankton to perform at a higher reaction rate and confer advantage through production of additional energy required to cope with exposure to toxic conditions. Therefore, we hypothesize that in addition to the specialized traits attained from the differences in genome composition, the ability of certain species to tolerate/resist the exposure to oxidative stress can be partly due to phenotypic plasticity of core metabolic pathways which allow them to perform at higher rates relative to sensitive species. To test this hypothesis, we used a chlorophyte that has been shown to be resistant to oil exposure, *Dunaliella tertiolecta* UTEX LB 999, as a model phytoplankton species (Bretherton et al. 2018). Using metabolic inhibitors, we blocked multiple core metabolic pathways such as PET, CET of PSI, Kreb's cycle, MET, PPP, and photorespiration and exposed the *D. tertiolecta* to water accommodated fraction (WAF) of oil. WAF is the most commonly used approach to obtain saturated hydrocarbon aqueous samples for oil toxicity testing (Forth et al. 2017). We then identified the metabolic pathway that conferred their oil‐tolerating ability and confirmed the role of the identified crucial core metabolic pathways through further experimentation. Lastly, we determined the source of carbon and energy that allowed *D. tertiolecta* to maintain uncompromised growth in water accommodated fraction of oil.

## MATERIALS AND METHODS

### Materials

*Dunaliella tertiolecta* UTEX 999 culture was obtained from UTEX. The inhibitors used in this study 3‐(3,4‐dichlorophenyl)‐1,1‐dimethylurea (DCMU; Catalog no: D2425), 2,5‐dibromo‐3‐methyl‐6‐isopropyl‐p‐benzoquinone (DBMIB; Catalog no: 71993), sodium malonate (Catalog no: 63409), 6‐aminonicotinamide (Catalog no: A68203), antimycin A (Catalog no: A8674), and aminooxyacetate (AOA; Catalog no: C13408‐1G) were all purchased from Sigma‐Aldrich. The oil used for preparation was a Macondo surrogate oil provided by BP Oil.

### Experimental design

*Dunaliella tertiolecta* (UTEX 999) culture was maintained in f/2 medium under 60 μmol photons · m^−2^ · s^−1^ illumination 12:12 h light:dark cycle at a temperature of 19°C. Four experiments were carried out to address the hypothesis noted previoulsy. In the first experiment, metabolic inhibitors were used to identify the core metabolic pathway playing an important role in oil‐resisting ability of *D. tertiolecta*. In the second and third experiment, further analysis on the identified pathways, Kreb's cycle and photorespiration, were performed to explain how they were important during oil exposure. In the fourth experiment, we explored carbohydrate consumption in *D. tertiolecta* during oil exposure.

### Identification of crucial core metabolic pathways under oil exposure

*Dunaliella tertiolecta* in batch culture was exposed to Control (f/2 medium) and WAF for a period of seven days in triplicates. WAF was prepared by mixing sterile f/2 growth media and oil (400 μL · L^−1^). The solution was stirred overnight in the dark and then filtered through a 20 μm nylon mesh to exclude larger oil droplets. The resulting WAF had an oil concentration of 3.25 mg · L^−1^ measured as estimated oil equivalents according to Wade et al. (2011). Briefly, the hydrocarbons in the samples were extracted using dichloromethane, and the fluorescence was measured at 322/376 nm (excitation/emission) wavelength using a Shimadzu spectrofluorometer (RF‐5301PC; Shimadzu, Houston, TX, USA). To understand the oil‐resisting ability of *D. tertiolecta*, the cells were inoculated at a cell density of 10^5^ cells · mL^−1^ in control f/2 medium and WAF medium with and without metabolic inhibitors. The metabolic inhibitors used, and their respective target biochemical pathways are listed in Table 1.

**Table 1:** List of chemical inhibitors used for targeting the core metabolic pathways. All the inhibitors were used at a concentration of 300 μM.

Metabolic inhibitor	Target biochemical pathway
3‐(3,4‐dichlorophenyl)‐1,1‐dimethylurea (DCMU)	Photosynthetic electron transport (PET) between PS II and PS I (Hoch et al. 1963)
2,5‐dibromo‐3‐methyl‐6‐isopropyl‐p‐benzoquinone (DBMIB)	Cyclic and linear electron transport (CET) between PS II and PS I (Takano et al. 2015)
Sodium malonate	Kreb's Cycle (Fawaz and Fawaz 1962)
6‐Aminonicotinamide	Oxidative pentose phosphate pathway (PPP; Köhler et al. 1970)
Antimycin A	Mitochondrial electron transport (MET) chain (Kröger et al. 1973)
Aminooxyacetate (AOA)	Photorespiration (Kleczkowski et al. 1987)

### Growth and photo‐physiology of *Dunaliella tertiolecta*

The effects of exposure to WAF and/or metabolic inhibitors were monitored by measuring growth and photo‐physiology of *Dunaliella tertiolecta*. Chlorophyll fluorescence was monitored every day as a proxy of growth until the end of the experiment using a benchtop fluorometer (10 AU Fluorometer, Turner Designs, San Jose, CA, USA). Photo‐physiological measurements were carried out with the help of a pulse amplitude modulated fluorescence system (Phyto‐PAM, Walz, Germany) and FIRe fluorometer system (Satlantic, Halifax NS, Canada; Bretherton et al. 2018, 2020, Kamalanathan et al. 2019,  2021a). For both measurements, 10 mL of cultures were dark adapted for 15 min prior to measurements. The measurements with Phyto‐PAM provided parameters of relative electron transport rates (*r*ETR_max_), and light harvesting efficiency (*α*), whereas the FIRe fluorometer system provided parameters of maximum PSII quantum yield (*F*_v_/*F*_m_), σPSII (functional absorption cross‐section of PSII, Å^2^ per quanta), ρ (connectivity factor defining the excitation energy transfer between individual photosynthetic units), and τ (time constant of the relaxation kinetics of the fluorescence yield following the Single Turnover flash, μs).

### Kreb's cycle and oil exposure

The *Dunaliella tertiolecta* culture was exposed to Control (f/2 medium) and WAF at a cell density of 5 × 10^4^ cells · mL^−1^ in batch culture for a period of 48 h in triplicates. The role of Kreb's cycle on metabolism during oil exposure was confirmed through determination of respiration rates after 48 h time point. Respiration rates were measured using a Clark‐type oxygen electrode (Hansatech, Norfolk, UK), as per methods described in Kamalanathan et al. (2015, 2021a). Further confirmation of the upregulation of Kreb's/tricarboxylic acid (TCA) cycle was confirmed by measuring the concentration of cycle metabolites and rates of respiration. Briefly, cells of *D. tertiolecta* were collected after 48 h incubation in the Control and WAF treatment. For measuring the concentration of metabolites in the cells, 400 mL of cultures were filtered through GF/F membrane (0.7 μm, Whatman, United States) and 0.5 ppm of ^13^C‐citrate was added to the tubes as an internal standard and the metabolites were extracted using chilled 100% methanol. The methanol extract was then analyzed on LC–MS/MS for organic acid analysis.

### LC–MS analysis of organic acids

The LC–MS system comprised an Agilent 1260 UHPLC system (Agilent, Santa Clara, CA, USA) and triple‐quadrupole 6420 mass detector. Chromatographic separation of organic acids was achieved under isocratic conditions for 13 min per sample run using an Agilent Hi‐Plex H column (250 × 4.6 mm, 8 μm) heated to 50°C. The liquid mobile phase comprised 80/20 milli‐Q water/acetonitrile containing 0.01% formic acid, with a flow rate of 0.2 mL · min^−1^. Organic acids were detected in negative electrospray ionization (ESI‐) mode using nitrogen as the desolvation gas at 200°C (gas flow of 12 L · min^−1^) and capillary voltage at 3000 V. Organic acids were detected in single ion monitoring mode, and ions detected included: *m/z* (mass‐to‐charge ratio) 87 (pyruvic acid), *m/z* 89 (lactic acid), *m/z* 133 (malic acid), *m/z* 103 (hydroxybutyric acid), *m/z* 75 (glycolic acid), and *m/z* 195 (D4‐citric acid).

### Photorespiration and oil exposure

To confirm the role of photo‐respiration pathway in *Dunaliella tertiolecta*, the cells were inoculated at a cell density of 5 × 10^4^ cells · mL^−1^ in control f/2 medium and WAF medium with and without aminooxyacetate (AOA) in triplicates. Growth was measured microscopically by daily cell counts using a Neubauer hemocytometer. Further, incubation experiments with different concentrations of AOA were also performed, and the levels of reactive oxygen species were measured. Briefly, 10^5^ cells of *D. tertiolecta* were incubated in Control and WAF treatment with 0, 10, 50, 100, 200, and 300 μM of AOA. Cells (1 mL) were incubated with 10 mM of 2′,7′‐dichlorofluorescien diacetate (DCFH‐DA) for 60 min in the dark. The cells were centrifuged at 10,000*g* for 5 min. The supernatant was discarded, and the cells were resuspended with fresh f/2 media and the fluorescence in the cells were measured at 488/520 nm excitation/emission using BioTek Cytation™ 5 imaging reader controlled by Gen52.09 software (USA).

### Carbon and energy source(s) during oil exposure

A batch culture experiment was conducted with four treatments: *Dunaliella tertiolecta* cells were inoculated at a cell density of 5 × 10^4^ cells · mL^−1^ in control f/2 medium and WAF medium kept under 60 μmol photons · m^−2^ · s^−1^ illumination 12:12 h light:dark cycle and a parallel batch kept under complete darkness for a period of 48 h. Cells (50 mL) were then harvested by centrifugation at 1260*g* for 15 min, and the pellet was resuspended in 500 μL of MilliQ water. Total cellular carbohydrates were determined with Glucose as the standard using the 2,4,6‐Tris(2‐pyridyl)‐S‐triazine method (Myklestad et al. 1997) with minor modification by Hung et al. (2001). 100 μL of 0.7‐mM potassium ferricyanide was added to 100 μL of the resuspended pellet. The solution was then incubated for 10‐min at 100°C in the dark. After cooling, 100 μL of 2 mM ferric chloride and 200 μL of 2.5 mM 2,4,6‐Tris(2‐pyridyl)‐S‐triazine was added to the solution. The absorbance was then read at 595 nm after 30 min using a Cytation 5 imaging reader (BioTek) controlled by the software Gen5 v2.09 (BioTek).

Cellular lipid content was estimated through gravimetric analysis using methods described in Kamalanathan et al. (2015, 2021a). Briefly, 100 mL of the culture was centrifuged at 3000 rpm at for 15 min. The pellet was resuspended in 5:5:4 mL chloroform, methanol, and MilliQ water and incubated at 4°C overnight. After incubation, the samples were filtered through 25 mm pre‐combusted filter (Whatman GF/F) and the organic bottom layer was carefully transferred into a pre‐weighed screw‐capped bottle and dried under a gentle stream of N_2_. The bottle was reweighed after drying to determine the amount of total lipids.

Extracellular carbohydrate content of *Dunaliella tertiolecta* under control and WAF was estimated using affinity purified Concanavalin A (ConA) Lectin immobilized on microplates for ELISA assays (Shiu et al. 2020).

### Statistics

Student's *t*‐test or one‐way ANOVA with Tukey's test was performed using software package GraphPad Prism (v7.04; https://www.graphpad.com/scientific‐software/prism/).

## RESULTS

### Identification of crucial core metabolic pathways under oil exposure

**Fig. 1:**
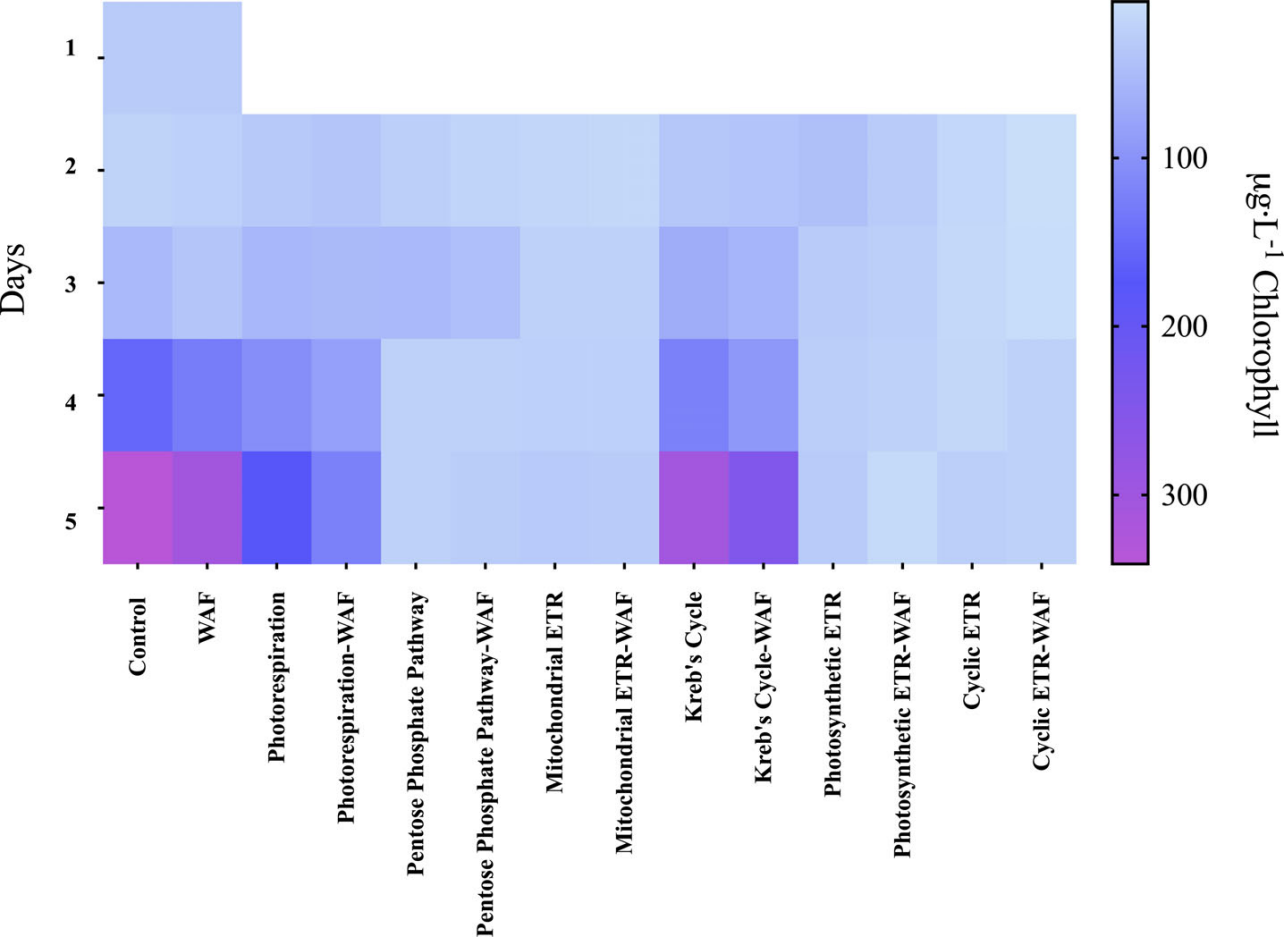
Heat map of chlorophyll *a* fluorescence depicting the growth response of *Dunaliella tertiolecta* in response to various metabolic inhibitors at 300 μM concentrations in the Control and WAF treatment (*n* = 3).

Chlorophyll *a* fluorescence was used as proxy of growth to measure the response of *Dunaliella tertiolecta* to various metabolic inhibitors in the Control and WAF treatment. Exposure of *D. tertiolecta* to WAF resulted in slight decrease in final biomass (~11%) compared with the Control (unpaired *t*‐test, *t*_4_ = 1.559, *P* = 0.1940; Fig. 1). Two different pathways were highlighted using various metabolic inhibitors, namely photorespiration and Kreb's cycle, that affected the growth of *D. tertiolecta* in WAF treatments relative to the corresponding controls (Fig. 1). Inhibition of photorespiration in WAF resulted in ~30% decrease in final biomass compared with the Control (unpaired *t*‐test, *t*_4_ = 13.02, *P* = 0.0002; Fig. 1). The growth rates were also significantly lower when photorespiration was inhibited in WAF (0.41 · d^−1^) compared with control (0.56 · d^−1^; unpaired *t*‐test, *t*_4_ = 6.485, *P* = 0.0029). Similarly, inhibition of Kreb's cycle resulted in ~20% decrease in final biomass in WAF compared with the Control treatment (unpaired *t*‐test, *t*_4_ = 2.444, *P* = 0.0288; Fig. 1). The growth rates of *D. tertiolecta* were significantly lower in WAF (0.6 · d^−1^) when Kreb's cycle was inhibited compared with the Control (0.711 · d^−1^; unpaired *t*‐test, *t*_4_ = 3.135, *P* = 0.0350). Inhibition of other pathways such as PPP, MET, PET, and CET alone resulted in no net growth in Control and WAF treatments.

**Fig. 2:**
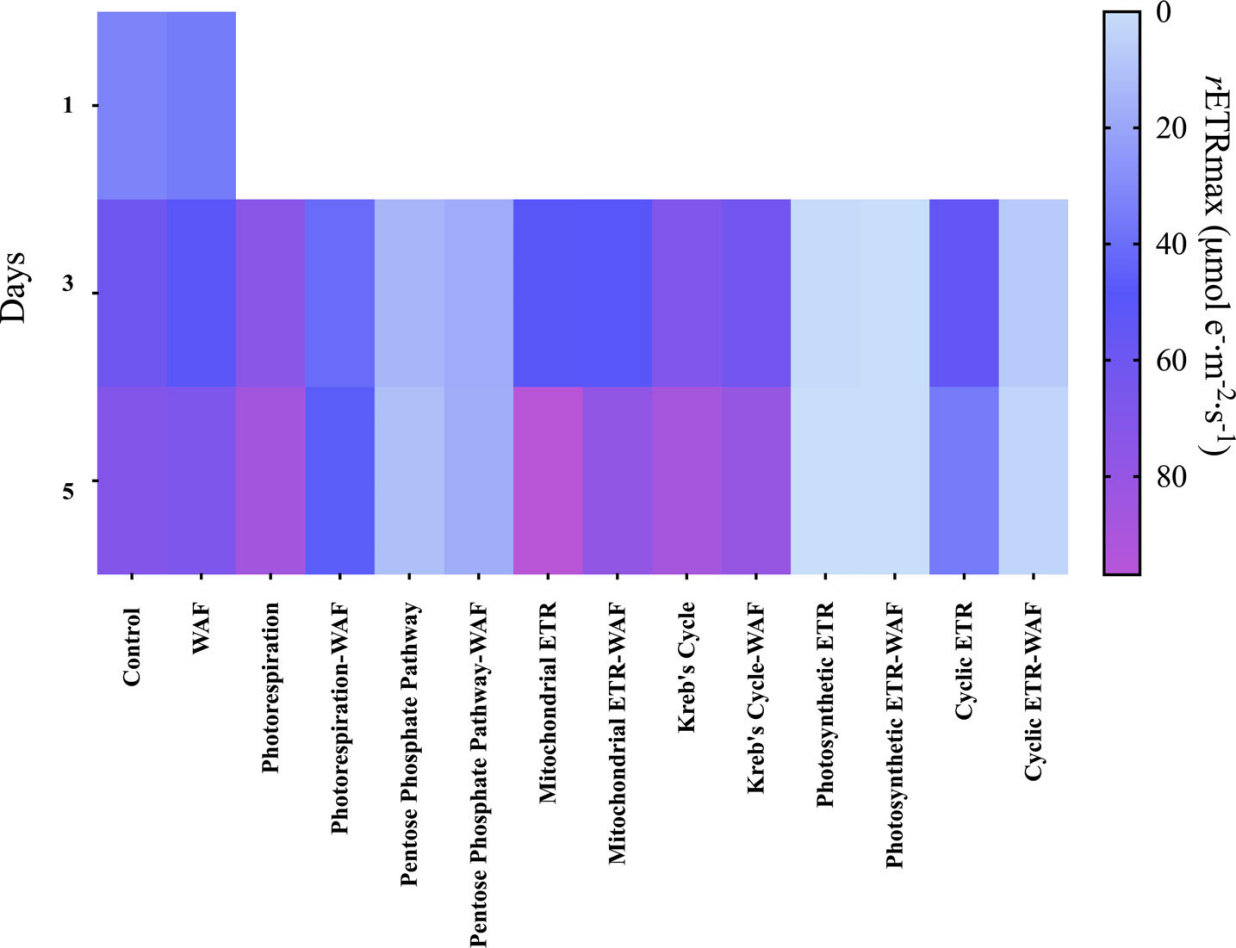
Heat map of maximum relative electron transport rates values (*r*ETR_max_) of *Dunaliella tertiolecta* in response to various metabolic inhibitors at 300 μM concentrations in the Control and WAF treatment (*n* = 3).

Along with growth, photosynthetic parameters of maximum quantum yield (*F*_v_/*F*_m_), relative electron transport rates (*r*ETR_max_) and light harvesting ability (*α*) were measured. *F*_v_/*F*_m_ measurements showed no significant differences between the treatments throughout the course of the experiment (Fig. S1 in the Supporting Information). *r*ETR_max_ measurements showed no significant difference between Control and WAF (unpaired *t*‐test, *t*_4_ = 1.502, *P* = 0.2076). However, when photorespiration was inhibited, the *r*ETR_max_ values were significantly lower (~42%) in WAF compared with the Control (unpaired *t*‐test, *t*_4_ = 3.626, *P* = 0.0222; Fig. 2). When Kreb's cycle, MET, and PPP was inhibited, *r*ETR_max_ values were not significantly different between WAF and the Control treatments (unpaired *t*‐test, *t*_4_ = 1.633, *P* = 1778; Fig. 2). However, inhibition of CET in WAF resulted in significantly lower (~86%) *r*ETR_max_ values compared with the Control (unpaired *t*‐test, *t*_4_ = 10.06, *P* = 0.0005; Fig. 2). Lastly, inhibition of PET resulted in complete inhibition of the electron transport as expected (Fig. 2).

### Kreb's cycle and oil exposure

**Fig. 3:**
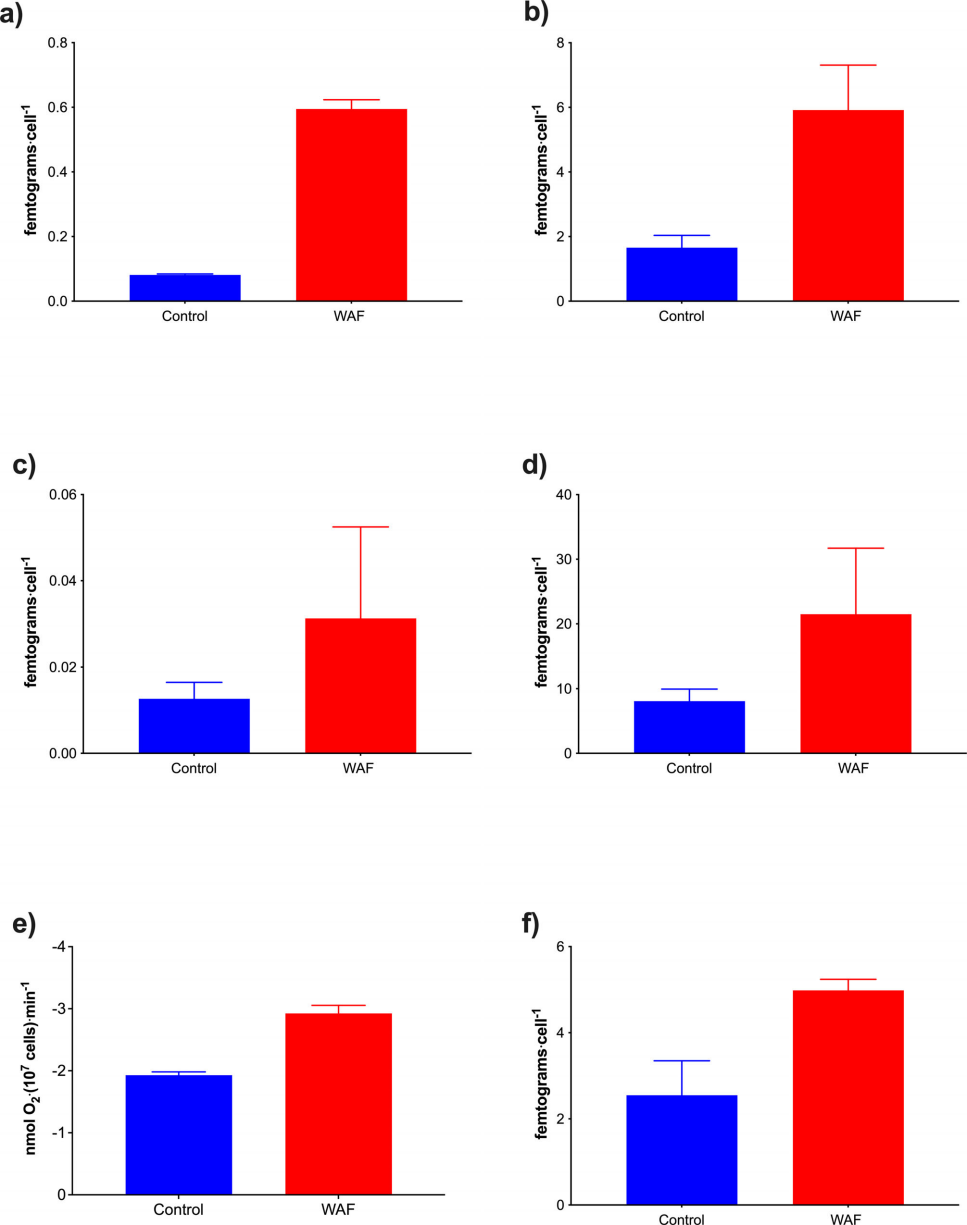
Metabolism of *Dunaliella tertiolecta* grown in Control and WAF (*n* = 3). Cellular concentrations of a, pyruvic acid (femtograms · cell^−1^); b, lactic acid (femtograms · cell^−1^); c, malic acid (femtograms · cell^−1^); d, hydroxybutyric acid (femtograms · cell^−1^); e, respiration rates (nmols O_2_ · cell^−1^ · min^−1^); and f, glycolic acid (femtograms · cell^−1^) in *D. tertiolecta* (*n* = 3).

To determine the role of Kreb's cycle during oil exposure, metabolite concentrations of central carbon metabolism and rates of respiration were determined after 48 h of incubation in Control and WAF. As oil concentration decreases below 1 mg · L^−1^ from 72 h onwards (Fig. S2 in the Supporting Information), a period of 48 h incubation was chosen as an optimal time point to monitor changes in metabolite levels. Concentrations of pyruvic acid and lactic acid were significantly higher (~86 and 71%) under WAF exposure compared with the Control (Pyruvic acid: unpaired *t*‐test, *t*_2_ = 25.24, *P* = 0.0016; lactic acid: unpaired *t*‐test, *t*_4_ = 5.124, *P* = 0.0069; Fig. 3, a and b). Similarly, levels of malic acid and hydroxybutyric acid were also higher under WAF exposure compared with the controls; however, these differences were not significant (Malic acid: unpaired *t*‐test, *t*_3_ = 1.612, *P* = 0.2053; hydroxybutyric acid: unpaired *t*‐test, *t*_4_ = 2.409, *P* = 0.0950; Fig. 3, c and d). Respiration rates, an indirect measure of flux through Kreb's cycle, was significantly higher (~34%) in WAF compared with the Control treatment (unpaired *t*‐test, *t*_4_ = 12.34, *P* = 0.0002; Fig. 3e).

### Photorespiration and oil exposure

**Fig. 4:**
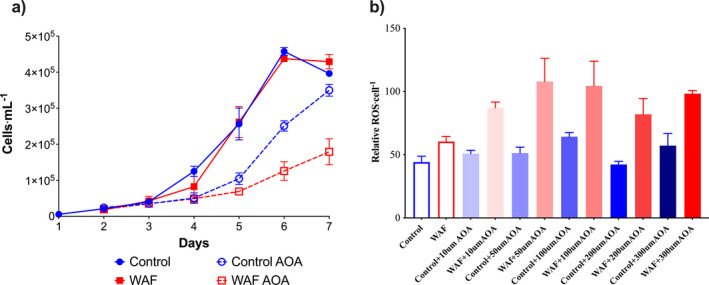
Role of photorespiration in *Dunaliella tertiolecta* under Control and WAF treatments. a, Cellular concentrations of *D. tertiolecta* in Control and WAF treatments in the presence and absence of photorespiration inhibitor aminooxyacetate at 300 μM (AOA; *n* = 3), b, Relative ROS levels · (cell^−1^) in *D. tertiolecta* under Control and WAF treatments with different concentrations of AOA (*n* = 3).

To determine the role of the photorespiration pathway during oil exposure, metabolite concentrations of glycolic acid were determined after 48 h of incubation in Control and WAF. Concentrations of glycolic acid were significantly higher (~48%) under WAF exposure compared to the Control (unpaired *t*‐test, *t*_3_ = 3.977, *P* = 0.0284; Fig. 3f). Furthermore, cell concentrations and metabolite concentrations were measured in the Control and WAF treatment in the presence and absence of photorespiration inhibitor aminooxyactate (AOA). Growth rates were similar between the Control and WAF treatment around an average of (0.034 · h^−1^; one‐way ANOVA, *F*_3,7_ = 0.329, *P* = 0.9323); however, in the presence of AOA, growth was relatively lower in both the treatment with 0.028 · h^−1^ in Control and 0.018 · h^−1^ in WAF (one‐way ANOVA, *F*_3,7_ = 0.329, *P* < 0.006; Fig. 4a). Moreover, the growth rates in WAF treated with AOA were significantly lower than the Control (one‐way ANOVA, *F*_3,7_ = 0.329, *P* = 0.0399; Fig. 4a).

Determination of reactive oxygen species (ROS) levels in the Control and WAF treatment revealed no significant differences (unpaired *t*‐test, *t*_3_ = 1.033, *P* = 0.3775; Fig. S2). As photorespiration has been hypothesized to prevent excess ROS accumulation and photo‐oxidative stress (Kozaki and Takeba 1996, Voss et al. 2013), we determined the role of photorespiration in regulating the intracellular ROS levels during oil exposure by inhibiting this pathway with different concentrations of inhibitor AOA. The ROS levels in the cells were slightly but not significantly higher in the WAF treatment compared with the Control (one‐way ANOVA, *F*_11,17_ = 1.111, *P* = 0.8676; Fig. 4b). While the ROS levels remained constant in the Control treatment at all concentrations of AOA (one‐way ANOVA, *F*_11,17_ = 1.111, *P* > 0.9809), the ROS levels in the WAF treatment *Dunaliella tertiolecta* cells increased when photorespiration was inhibited (Fig. 4b). ROS levels increased at 10 μM concentration of AOA (~48%), and significantly increased further at 50 (~78%; one‐way ANOVA, *F*_11,17_ = 1.111, *P* = 0.001), 100 (one‐way ANOVA, *F*_11,17_ = 1.111, *P* = 0.0263) and 300 μM (~62%) concentrations (one‐way ANOVA, *F*_11,17_ = 1.111, *P* = 0.022; Fig. 4b).

### Carbon and energy source during oil exposure

**Fig. 5:**
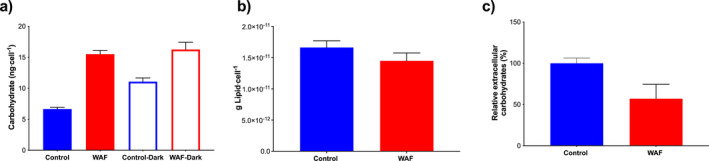
Macromolecular composition of *Dunaliella tertiolecta* in Control and WAF treatments. a, Cellular carbohydrate concentrations in *D. tertiolecta* in Control and WAF treatments under 12:12 h light:dark and 24 h dark treatments; b, cellular lipid concentrations; and c, extracellular carbohydrate concentrations in *D. tertiolecta* in Control and WAF treatments (*n* = 3).

To understand the source of carbon and energy during oil exposure, we determined the cellular carbohydrate and lipid content during oil exposure after 48 h of incubation. Estimation of cellular carbohydrate content revealed significantly higher levels (~53%) in WAF compared with the Control (one‐way ANOVA, *F*_3,4_ = 3.394 E+029, *P* = 0.0014; Fig. 5a). Similarly, the carbohydrate content was higher (~31%) under complete dark incubation in WAF compared with the 12:12 h light:dark cycle incubated Control (one‐way ANOVA, *F*_3,4_ = 3.394 E+029, *P* = 0.0012), however the values were not significantly different from 12:12 h light:dark cycle incubated WAF (one‐way ANOVA, *F*_3,4_ = 3.394 E+029, *P* = 0.3650; Fig. 5a). A similar significant boost in cellular carbohydrate content was observed in the dark Control treatment relative to the 12:12 h light:dark cycle Control (~39%; one‐way ANOVA, *F*_3,4_ = 3.394 E+029, *P* = 0.0115) and WAF (~11%; one‐way ANOVA, *F*_3,4_ = 3.394 E+029, *P* = 0.0115) counterpart (Fig. 5a). Estimation of cellular lipid showed no significant differences between the Control and WAF treatment (unpaired *t*‐test, *t* = 1.827, *P* = 0.2092; Fig. 5b). However, the determination of extracellular carbohydrate content showed significantly lower levels (~43%) in WAF compared with the control treatment (unpaired *t*‐test, *t* = 4.004, *P* = 0.0161; Fig. 5c).

## DISCUSSION

Phenotypic plasticity allows an organism of a single genotype to produce distinct phenotypes in different environments (Pigliucci 2001) without requiring genetic alterations (Price et al. 2003). While specialized traits attained because of the differences in genome composition can certainly provide selective advantage in variable environments (Van Der Meer et al. 1992, Barrett and Schluter 2008, Ritter et al. 2010), plasticity in phenotype can also play a significant role (Nussey et al. 2005, Suzuki and Nijhout 2006, Ghalambor et al. 2007, Dey et al. 2016). It is of particular interest when a rapid environment‐altering event occurs, such as an oil spill, to follow the differential responses of members of the phytoplankton community. While some find the oil toxic, others continue to grow and photosynthesize albeit at their regular pace (e.g., Bretherton et al. 2018, 2020). In the case of oil spills, it has been noted that toxin production may be triggered (Bretherton et al. 2019). Long‐term incubation studies by Carrera Martinez et al (2010) and Romero‐Lopez et al. (2012) highlighted the importance of rare spontaneous mutations in the development oil‐tolerating ability in species like *Dunaliella tertiolecta*, *Scenedesmus intermedius*, and *Microcystis aeruginosa*. However, over a short‐term incubation with no prior acclimation that better reflect the instant exposure effect caused during an oil spill, *D. tertiolecta* has been shown to tolerate oil exposure through changing pigment composition such as increasing β‐carotene: chlorophyll and lutein:chlorophyll and cellular phenolic content (Salinas‐Whitaker et al. 2020). Such changes in cellular pigment and macromolecules in turn help in quenching singlet oxygen (Tammam et al. 2011) and dissipating excess absorbed light energy (Salinas‐Whitaker et al. 2020). While this and other cellular changes are often investigated, less known is the role of phenotypic plasticity in core metabolic pathways in conferring uninhibited growth during exposure to oil spill.

Growth measurements under the presence of inhibitors targeting specific core metabolic pathways suggested that both Kreb's cycle and photorespiration played a crucial role in conferring resistance to the chlorophyte *Dunaliella tertiolecta* against oil exposure. Interestingly, no net growth was observed when the CET was inhibited. Linear photosynthetic electron transport values in WAF were lower compared with controls which suggests that *D. tertiolecta* was alive and dormant. It also underscores the crucial role CET might play during oil exposure. Rumeau et al. (2007) had previously suggested that CET might supply additional ATP during stressful conditions, while Munekage et al. (2004) has shown that this pathway can help protect against stromal overreduction. The lower linear electron transport values in WAF compared with controls also indicate the possibility of higher stromal overreduction in WAF when CET was inhibited, consistent with the observed lower light harvesting capacity in WAF (Unpaired *t*‐test, *t*_4_ = 4.450, *P* = 0.0112; Fig. S3 in the Supporting Information). The latter suggests oil‐induced damage to the photosynthetic apparatus. Such damage to light harvesting capacity has been previously observed under oil exposure in diatoms (Kamalanathan et al. 2021a). However, whether the energy demand of cells was higher during oil exposure remains to be determined as does the role of CET in supplying the additional ATP.

The overall higher metabolite concentrations of pyruvic acid, malic acid, and hydroxybutyric acid along with significantly higher respiration rates under oil exposure (and the growth measurements) collectively confirm the vital role of Kreb's cycle and suggests the possibility of higher carbon and energy flux through this pathway, and in general aerobic metabolism in *Dunaliella tertiolecta*. Such an increase in Kreb's cycle metabolites has been observed previously for *Nitzschia closterium* and *Heterosigma akashiwo* also in response to oil exposure (Li et al. 2019). Effects of stress on Kreb's cycle and aerobic respiration has been shown to vary, with over expression observed in some species such as *Karenia mikimotoi* (Shi et al. 2021), *Nannochloropsis oceanica* (Wang and Jia 2020), *Phaeodactylum tricornutum* (Matthijs et al. 2017), and inhibition in others such as *Isochrysis galbana* (Su et al. 2017), *Phaeocystis* sp. (Stefels and van Leeuwe 1998). This highlights the range of phenotypic plasticity of this pathway among different species of phytoplankton and helps explain the differential response patterns observed. Based on this broad literature, we hypothesize the over induction of aerobic metabolism in *D. tertiolecta* could be indicative of higher energy demands during oil exposure. Supporting this, H_2_O_2_‐induced oxidative stress can influence glycolysis and mitochondrial function, suggesting that cellular energy demands are met under stress by dynamic regulation of mitochondrial activity (Hyslop et al. 1988). However, significantly higher metabolite concentrations of lactic acid were also observed under oil exposure, which suggests the likelihood of an onset of mixed respiro‐fermentative metabolism. Considerable amounts of ROS can also form as a result of interaction between oxygen and the reduced form of flavins and ubiquinone in the MET (Møller 2001). We hypothesize that respiro‐fermentative metabolism under oil exposure can be a potential mechanism in *D. tertiolecta* to balance ROS production while meeting the higher energy demands.

The overall higher metabolite concentrations of glycolic acid were significantly higher under oil exposure, which along with the growth measurements suggest a crucial role of photorespiration. The physiological function of photorespiration since its discovery in 1920 (Warburg 1920) remains unclear, with only suggestive functions (Moroney et al. 2013, Hagemann et al. 2016). Often viewed as unfavorable to primary productivity, photorespiration is widely considered to be a wasteful process (Wingler et al. 2000, Busch 2020). Factors such as (i) the retention of oxygenation, (ii) the lack of improvement to fix CO_2_ by RuBisCO despite the present‐day lower concentrations of CO_2_ (relative to the time when RuBisCO evolved) in the atmosphere, and (iii) the inefficient growth among mutant species defective in photorespiratory enzymes (Eisenhut et al. 2008, Zelitch et al. 2009), suggests that photorespiration must have a fundamentally essential function. Photorespiration has been previously hypothesized to prevent excess ROS accumulation and photo‐oxidative stress (Kozaki and Takeba 1996, Voss et al. 2013). The observed increasing cellular ROS levels during oil exposure under increasing inhibition of photorespiration confirms anti‐oxidation as one of the physiological functions. In addition, several studies have shown that the glycolate secreted by *Dunaliella tertiolecta* and other species of *Dunaliella* is then reassimilated in the cells (Nimer et al. 1990, Sciandra et al. 1997, Leboulanger et al. 1998), through the action of glycolate oxidase or glycolate dehydrogenase into the glyoxlate pathway (Nimer et al. 1990, Stabenau et al. 1993). Moroney et al. (2013) suggested photorespiration “limits” the loss of carbon by oxygenation by RuBisCO, as only one molecule of CO_2_ is lost after accounting for glycolate oxidase or glycolate dehydrogenase activity. Therefore, even though photorespiration is currently considered a wasteful process, the findings in this study suggest that it allows cells to counteract oxidative stress while minimizing the loss of carbon and energy fixed. The oxygen consumed during the photorespiration might help in creating a more reduced environment in the cell, thereby decreasing the extent of ROS formation. Lastly, we also observed reduced photosynthetic electron transport under oil exposure when photorespiration was inhibited, confirming a crucial role of this pathway in maintaining photosynthesis as previously suggested (Munekage et al. 2004).

While the above findings underscore the importance of metabolic plasticity in Kreb's cycle and photorespiration in surviving oil exposure, it does not explain how *Dunaliella tertiolecta* was able to maintain uncompromised growth compared with the control treatment. No difference in cellular lipids and higher levels of carbohydrates under oil exposure suggest that increased catabolism of internal carbon reserve may not be providing the additional carbon and energy demands. Moreover, given the significant increase in carbohydrates was observed in the control under dark incubation while no such change occurred during oil exposure, indicate a decreased consumption of this internal carbon reserve. Previous studies have shown significant excretion of polysaccharides/carbohydrates by various phytoplankton (Myklestad 1995, Hama and Yanagi 2001) including several species of *Dunaliella*. Such secretion of carbohydrates can amount up to 80–90% of the total photosynthate exudate (Myklestad 1995). Decreased levels of extracellular carbohydrates in oil exposed *D. tertiolecta* (1.75‐fold lower) compared with Control suggest that the cells might be conserving carbon and energy by at least ~40% through reducing the “loss” through such active secretion. Such conservation of carbon and energy might explain how *D. tertiolecta* were able to maintain uncompromised growth during oil exposure. However, extracellular secretion by phytoplankton has been shown to play a significant role in phytoplankton–microbial interactions (Kamalanathan et al. 2021b). Therefore, despite oil exposure having no net effect on the growth of *D. tertiolecta*, it might have ecological consequences.

Overall, the crucial roles of CET, Kreb's cycle, and mitochondrial respiration in *Dunaliella tertiolecta* during oil exposure suggests that the energy demands of the cells were relatively higher. The antioxidant role of photorespiration pathway during oil exposure not only explains the growth response of *D. tertiolecta* during exposure to oxidative‐stress‐inducing pollutant such as oil but also offers cues to one if not all the obscurely known physiological roles of photorespiration. It is important to note that our approach of using inhibitors has limitations due to its non‐specific mode of action, for example, even though AOA inhibits photorespiration through impacting glycollate metabolism (Jenkins et al. 1983), it can also induce amino acid accumulation (Brunk and Rhodes 1988). Similarly, sodium malonate used in our study for inhibiting Kreb's cycle (Price 1953) can also impact nitrogenase (Murry et al. 1984), which is not present in *D. tertiolecta*. However, the role of crucial pathways highlighted from metabolic inhibitor experiments were also confirmed through measurements of respective metabolite levels and other physiological measurements to support the findings. In conclusion, using metabolic inhibitors and analytical approaches, our study indicates how phenotypic plasticity of multiple core metabolic pathways (such as Kreb's cycle, mitochondrial respiration, photorespiration, and EPS secretion in *D. tertiolecta*) together with the cellular pigment and macromolecular changes (in *D. tertiolecta*) shown by Salinas‐Whitaker et al. (2020) can account for the oxidative stress (through immediate oil exposure in this study) tolerating ability of certain phytoplankton species.

## AUTHOR CONTRIBUTIONS

**M. Kamalanathan:** Conceptualization (lead); data curation (lead); formal analysis (lead); investigation (lead); methodology (lead); project administration (equal); software (lead); supervision (lead); validation (lead); visualization (lead); writing – original draft (lead); writing – review and editing (equal). **S. Mapes:** Methodology (equal); writing – review and editing (equal). **A. Prouse:** Methodology (equal); writing – review and editing (equal). **P. Faulkner:** Methodology (equal); writing – review and editing (equal). **N.H. Klobusnik:** Methodology (equal); writing – review and editing (equal). **J. Hillhouse:** Data curation (equal); methodology (equal); writing – review and editing (equal). **D. Hala:** Methodology (equal); writing – review and editing (equal). **A.S. Quigg:** Funding acquisition (lead); project administration (equal); resources (lead); supervision (equal); writing – review and editing (equal).

## Supporting information


**Figure S1**. Heat map of maximum quantum yield (*F*_v_/*F*_m_) values of *Dunaliella tertiolecta* in response to various metabolic inhibitors at 300 μM concentrations in the Control and WAF treatment (*n* = 3).

**Figure S2**. Relative ROS levels · (cell^−1^) in *Dunaliella tertiolecta* under Control and WAF treatments (*n* = 3).

**Figure S3**. Light harvesting capacity (α; μmol e^−1^ · μmol photons^−1^) in *Dunaliella tertiolecta* under Control and WAF treatments when CET was inhibited (*n* = 3).
