# Supplementary figures and images for: Core metabolism plasticity in phytoplankton: Response of Dunaliella tertiolecta to oil exposure
Source: J Phycol. 2022 Sep 29;58(6):804–14. doi: 10.1111/jpy.13286 (PMC10087180; doi:10.1111/jpy.13286)

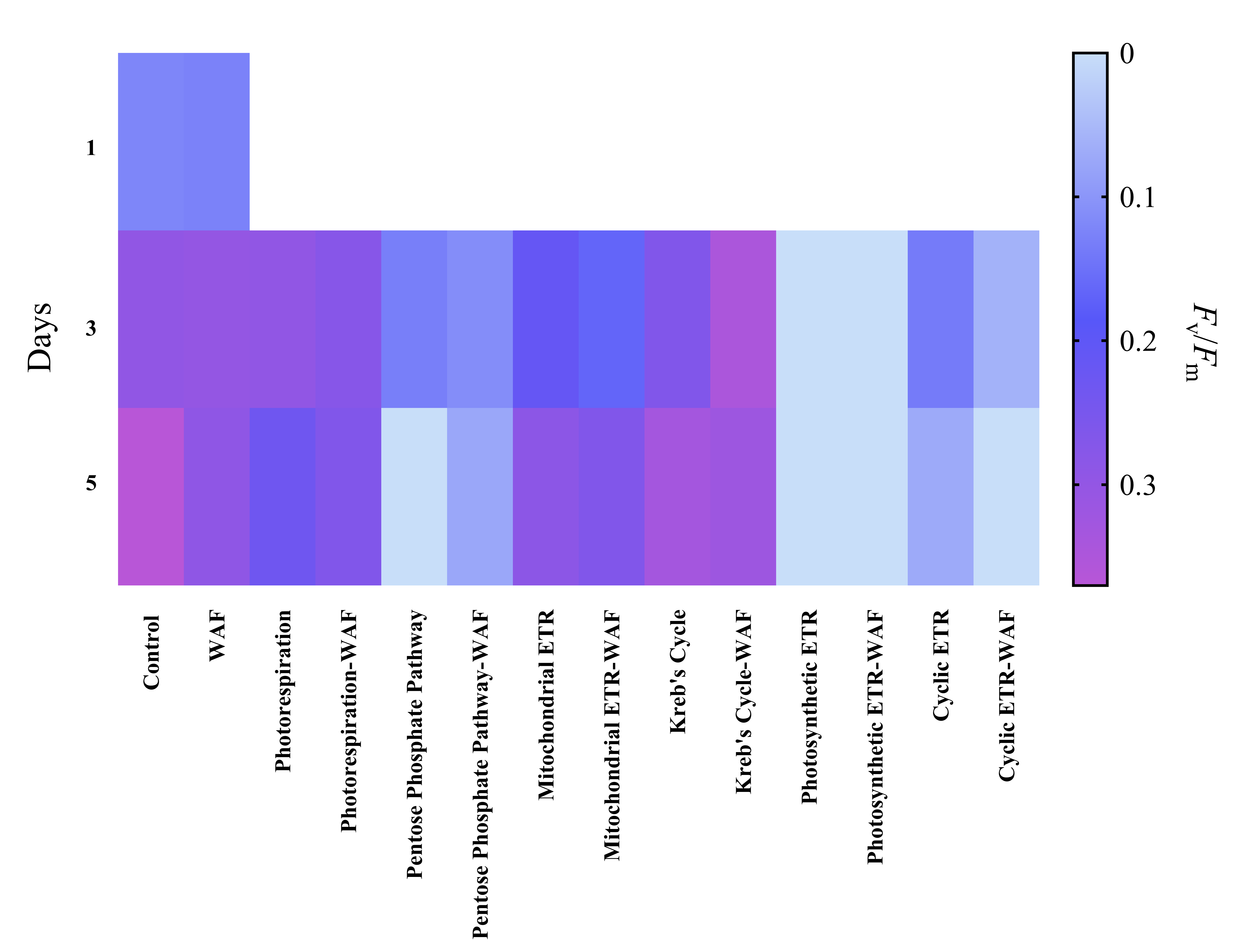

Supplement: Supplementary file 1 — Figure S1. Heat map of maximum quantum yield (F v/F m) values of Dunaliella tertiolecta in response to various metabolic inhibitors at 300 μM concentrations in the Control and WAF treatment (n = 3). [file JPY-58-804-s001.tif]

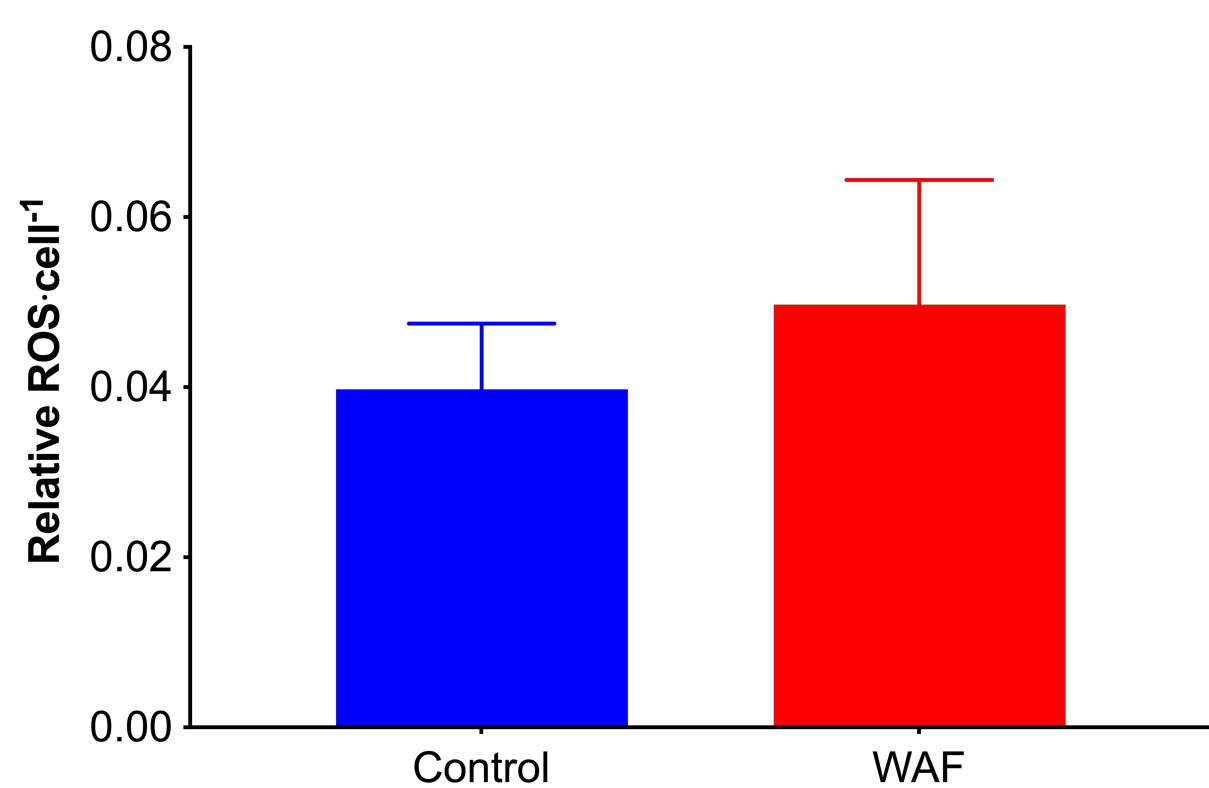

Supplement: Supplementary file 2 — Figure S2. Relative ROS levels · (cell−1) in Dunaliella tertiolecta under Control and WAF treatments (n = 3). [file JPY-58-804-s002.pdf]

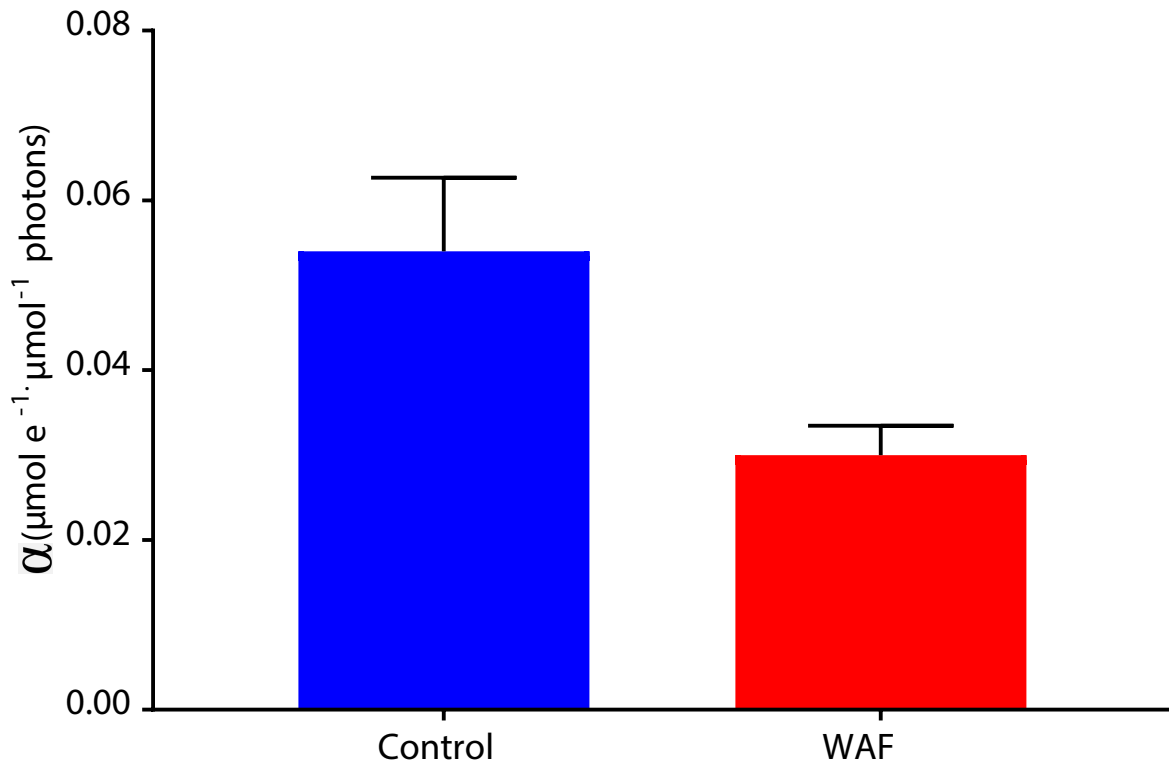

Supplement: Supplementary file 3 — Figure S3. Light harvesting capacity (α; μmol e−1 · μmol photons−1) in Dunaliella tertiolecta under Control and WAF treatments when CET was inhibited (n = 3). [file JPY-58-804-s003.pdf]
